# The Regional Implementation of an Electronic Health Record–Integrated Ambient Scribe in Primary and Secondary Care in England: Real-Time Qualitative Evaluation

**DOI:** 10.2196/88472

**Published:** 2026-05-29

**Authors:** Kathrin Cresswell, Catharine Rose, Jessica Howdle, Lucas Martinus Seuren, Robin Williams

**Affiliations:** 1 Usher Institute, University of Edinburgh, 5-7 Little France Road, Edinburgh BioQuarter—Gate 3, Edinburgh, EH16 4UX, Scotland, 44 131 651 7869; 2 Evaluation Hub, University of Worcester, Worcester, England, United Kingdom; 3 Institute for the Study of Science, Technology and Innovation, University of Edinburgh, Edinburgh, Scotland

**Keywords:** ambient scribes, note-taking, sociotechnical, qualitative, process evaluation, integration, health care

## Abstract

**Background:**

There is significant potential for ambient scribe technology to enhance health care productivity, with a growing range of applications being developed and implemented internationally. Strong organizational drivers to improve efficiency, coupled with the technology’s potential to help address clinician burnout, are accelerating interest and adoption. However, limited attention has been paid to the integration of such systems within electronic health records and unintended consequences, as stakeholders navigate their implementation and integration into clinical practice.

**Objective:**

This study therefore aimed to explore the processes involved in implementing and adopting ambient scribe technology across diverse health care settings.

**Methods:**

We conducted a real-time, longitudinal qualitative evaluation of a pilot implementation of an integrated ambient scribe system across National Health Service primary care and secondary hospital settings within a care system in the Midlands region of England. Data collection involved in-depth one-to-one interviews conducted in several phases: an initial scoping study to identify key interests and stakeholders for inclusion, followed by an implementation study with participants involved in the pilot. We also conducted 16 hours of nonparticipant observations of consultations. The implementation study gathered data at 2 time points, before implementation and 3 to 4 weeks after, to capture experiences, changes, and emerging impacts over time.

**Results:**

We collected data from 45 individuals. Use cases varied across settings and shaped how ambient scribe systems were deployed. Differences between general practice and secondary care in documentation purpose, format, and workflow created challenges for developing and validating templates, though the technology showed flexibility across contexts. Automated note-taking often improved patient interaction but required clinicians to adjust how they spoke to ensure the technology captured their reasoning. Outputs were sometimes generic, reinforcing defensive documentation and reducing personal tone or contextual recall. Integration pathways carried distinct trade-offs: stand-alone systems (which were used by many stakeholders in our study) were easier to adopt but offered limited long-term benefits, while integrated systems required greater effort and standardization, yet promised improved efficiency and safety.

**Conclusions:**

The ambient scribe market remains immature and volatile, creating strategic uncertainty for health systems. Careful procurement approaches are needed to balance the risks and benefits of integrated versus stand-alone systems, while aligning user demand with organizational needs for integration.

## Introduction

With aging populations and rising prevalence of long-term conditions and patient complexity, health care systems are under growing pressure to improve the efficiency of care delivery [1,2]. Ambient artificial intelligence (AI) scribes offer substantial potential to enhance organizational efficiency, alleviate health care professional burnout associated with electronic data entry, and improve the quality of patient-provider relationships [3,4]. These tools do not merely transcribe speech, but they can also produce various kinds of summaries (eg, for patient letters, referrals, and discharge letters). If they are integrated, they can also populate electronic health records (EHRs). See Table 1 for definitions.

**Table 1.** Definitions of terms.

Term	Definition
Ambient voice technology (AVT)	Infrastructure (eg, microphones, voice detection, and speaker diarization) that detects, separates, and prepares speech streams for processing.
Machine learning	Data-driven methods in which models learn mappings from data, including supervised, unsupervised, and reinforcement learning.
Natural language processing (NLP)	Branch of artificial intelligence concerned with the computational processing of human language in text form; used to transform transcripts into structured notes.
Large language model (LLM)	Neural language model trained on large corpora that transforms consultation transcripts into summaries and structured electronic health record (EHR) notes.
Digital scribe	System that records consultations and uses automatic speech recognition (ASR) and NLP models to extract or summarize relevant clinical information.
Ambient artificial intelligence scribe (ambient scribe)	System that records consultations using AVT, ASR, and LLMs to extract clinical information, generate summaries, and populate EHRs.

Clinicians currently spend a considerable amount of time on tasks such as note-taking during consultations, updating EHRs after consultations, and generating summary reports (eg, to facilitate referral or discharge letters) [5]. The effort of data capture for EHRs, compounded by their complexity and uneven usability, has consistently been found to be a contributing factor to stress and burnout [6-8]. By automating note generation, ambient scribes have the potential to reduce data entry work and improve job satisfaction [9,10]. They can also facilitate generating summary reports for various purposes, including referrals and discharge letters, thereby speeding up reporting [11].

Emerging evidence on the benefits of ambient scribes through improvements in efficiency and patient-provider relationships is also encouraging. For example, a 5-week trial with a diversity of clinicians (mainly family medicine) in Pennsylvania found that ambient scribe use was associated with a 30% reduction in daily after-hours work and 20.4% less time spent on notes during consultations (from 10.3 to 8.2 minutes; *P*<.001) [9]. Similarly, a prospective study across a range of clinical settings (mainly primary care) at Stanford Health found that ambient scribe users spent less total daily time (19.95 minutes) and after-hours time (5.17 minutes) maintaining EHRs [10]. In the United Kingdom, a multisite study on ambient scribe use conducted across hospital, primary, and mental health settings reported a 28% increase in time spent on direct patient care during consultations as well as an 8.2% reduction in consultation length (from 18.4 minutes at baseline to 16.9 minutes) [12,13].

These early benefits have attracted numerous vendors, many of whom are aggressively marketing products directly to clinicians, for example, by offering free use to build support ahead of expected procurement. However, there has been an increasing consideration of potential risks and unintended consequences associated with ambient scribes [14]. This pattern reflects a familiar dynamic, akin to the Gartner “hype cycle” [15], in which initial enthusiasm and inflated expectations are followed by a period of disappointment before a more balanced understanding of real-world benefits and limitations emerges.

Growing concerns about potential risks have prompted calls for stronger regulation and oversight [16-19]. For example, one key motivation for integrating ambient scribes is to enhance the accuracy of clinical records [20], which are often compromised by errors such as incompleteness, inaccuracy, and inconsistency [21,22], all of which can lead to patient harm [23]. However, emerging evidence suggests that ambient scribes may introduce new types of documentation errors (eg, hallucinations and omissions) that also have the potential to lead to considerable patient harm [24,25]. Furthermore, some clinicians report that notes generated by ambient scribes can be of lower quality than those composed by humans, often using simplistic language and lacking clarity or detail [9,26]. Consequently, some clinicians still spend considerable time editing generated notes [9,27].

Unintended consequences often emerge only during real-world use, as adopters and implementers integrate technological functionality into routine practice within their specific settings [28]. Ambient scribe technology is advancing rapidly, driven both by improvements in core technologies and by their adaptation to specific health care processes over time. In parallel, the outcomes we achieve with these tools are also evolving, as organizations gain experience, develop new skills, and optimize their use.

This evolution presents challenges for traditional evaluation strategies, such as randomized controlled trials of standardized interventions. Consequently, there is a growing need for rapid, real-time assessment and learning to appropriately balance benefits and risks [29].

Overall, existing evaluations of ambient scribes have predominantly focused on short-term pilot implementations, efficiency metrics, and clinician satisfaction outcomes. There has also to date been limited attention to unintended organizational and workflow consequences and integration challenges within existing EHR ecosystems.

We therefore here aimed to explore the processes involved in implementing and adopting an ambient scribe intended to be integrated within existing EHRs across diverse primary and secondary health care settings spanning 2 English counties.

## Methods

### Design

We conducted a real-time longitudinal qualitative evaluation of a pilot implementation of an integrated ambient scribe system in a range of National Health Service (NHS) primary care and secondary hospital-based settings within a care system in the Midlands region of England. The organization launched the pilot between May and September 2025 to explore the potential benefits of scribe technology integrated directly with EHRs. This came at a time when many clinicians were informally using nonintegrated tools and manually copying and pasting outputs into the EHR, creating workflow and safety risks. The evaluation was undertaken between January and October 2025.

### Ethical Considerations

Ethics approval was granted by the Health and Sciences Research Ethics Panel, University of Worcester (reference HS24250031-R). Participants received an information sheet and consent form in advance of the interview, which was arranged at a time convenient for the participant. Participants did not receive any financial compensation or other reimbursement for their participation in this study. They were free to withdraw at any time, and all provided written informed consent to participate before the interview was conducted. All identifiable information was removed from the interview recordings and transcripts, and findings were collated and summarized to protect participants’ identities.

### Study Setting

The care system we studied is responsible for coordinating health and social care across 2 Midlands counties in England. It brings together the NHS, local government, community health providers, and voluntary organizations to plan and deliver services jointly and to improve the overall quality and efficiency of care for the local population. Study settings included rural communities, market towns, and 2 outpatient clinical specialties in a city hospital based in a predominantly rural county. The vendor granted pilot sites a limited number of temporary licenses for the ambient scribe for the study period. A second ambient scribe system was being piloted at the same time in secondary care outpatient settings. This was not the primary focus of this evaluation, but participants reported on its use, so it emerged as an important aspect of the study. Some clinicians involved in this study had used, or previously had experience with, both systems. No other ambient scribe system was in use among the participating primary care staff. However, all described themselves as confident technology users who were open to the potential of AI. They also noted that their colleagues and peers had reported using ambient scribes with varying degrees of success. The role of the ambient scribe in the clinical interaction is described in Textbox 1.

Textbox 1. **The role of the ambient scribe in the clinical interaction.**

Across study sites, ambient scribing systems were used in 2 configurations: an integrated system embedded within the electronic health record (EHR) and accessed via the clinician’s desktop computer and a stand-alone system accessed via a mobile phone, requiring manual copying of information into the EHR. Despite these technical differences, the implications for patient-clinician interaction were broadly similar.

At the start of the consultation, clinicians typically greeted the patient and then sought explicit consent to use the ambient scribe. If consent was granted, the clinician activated the recording function. Clinicians then switched the recording function off when the patient had left the room or paused recording to seek consent from the following patient before switching the recording system back on. During the consultation, clinicians generally refrained from typing and instead focused on interacting with the patient, while ensuring that the computer or phone was positioned close enough to capture the conversation. At the conclusion of the clinical encounter, the clinician deactivated the scribe and reviewed the generated output. This review process involved checking accuracy, making corrections where necessary, and, in the case of the stand-alone system, manually copying and pasting relevant information into the patient’s electronic record.

### Sampling and Recruitment

The study used 2 complementary sampling strategies associated with 2 different parts of the study.

The first scoping phase involved qualitative, semistructured interviews with a range of professional stakeholders, including vendors, managers, and clinicians who were involved in developing the strategy and were among the early users of ambient scribes. Stakeholders were identified either by the project management team or through recommendations from those initially interviewed, as we expanded the sample through snowball methodology. Each potential participant was independently contacted by a member of the research team (KC), who invited them to participate and provided a participant information pack and consent form. This purposive sample was designed to capture diverse perspectives from individuals involved in the strategic development and decision-making surrounding the use of ambient scribes in both primary and secondary care. It also informed the topic guides of the implementation study. Recruitment for this phase concluded when the interviewers (KC and RW) determined that a sufficiently varied and representative group of stakeholders had been included, reflecting a broad range of experience, knowledge, and roles. Three participants were interviewed twice, once at the beginning and again at the end of the study, to explore evolving insights and lessons learned over time.

The second part of the study focused on user experiences. We invited all clinicians and medical secretarial staff in both primary and secondary care who were participating in the pilot to take part in evaluation interviews. Two members of the research team (CR and JH) were provided with participants’ email addresses and workplace details by the pilot project management team and contacted them directly to invite participation. Each was provided with a participant information sheet and consent form. No interviews or observation sessions were conducted until participants had given written consent and confirmed that they had read the information sheet.

### Data Collection

Data collection consisted of two phases: (1) stakeholder mapping and scoping interviews before the pilot and (2) an implementation study of the pilot, involving baseline and follow-up data collection (Table 2).

**Table 2.** Overview of data collection activities.

Phase of the work	Data collection method	Participants	Location
Stakeholder mapping and scoping	Semistructured interviews with stakeholders to identify interests, needs, expectations, and insights for the evaluation.	Key stakeholders identified through gatekeeper recommendations, including staff and ambient scribe vendors involved in management, procurement, or decision-making.	Online
Stakeholder mapping and scoping	Review of relevant documents provided by stakeholders (eg, business cases, strategies, meeting minutes, and development plans).	Key stakeholders identified through gatekeeper recommendations, including staff and ambient scribe vendors involved in management, procurement, or decision-making.	Not applicable
Baseline data collection from pilot sites	Semistructured qualitative interviews and real-time observations exploring current use, workplace processes, expectations, concerns, and barriers.	Primary and secondary care teams involved in the pilot; clinicians and medical secretarial staff.	Regional sites; interviews online or onsite; observations onsite
Follow-up data collection from pilot sites	Semistructured interviews to explore implementation and adoption experiences and follow up on baseline topics.	Participants from baseline plus additional clinicians or medical secretarial staff involved in the pilot.	Online or onsite (clinical workplace)

Stakeholders and users of the ambient scribe system under study were interviewed before the pilot began (baseline), and participants in the implementation study were followed up 4 to 5 weeks later. Observational data were also collected by the research team at a subset of implementation sites during baseline and follow-up phases to support understanding of the context and approaches to clinical note-taking across these settings, both with and without the use of ambient scribes. Observations took place during clinical consultations and were nonparticipant in nature (conducted by CR and JH). They involved researchers noting down details surrounding location, setting, equipment, participants, activities, and researcher impressions.

Interviews were conducted either over Microsoft Teams or in a private space at the participant’s workplace. Topic guides for the interviews were developed collaboratively through discussion with the research team and the pilot management team (Table 3). They were informed by prior literature on digital health implementation and sociotechnical systems and structured around key domains of the technology-people-organization-macroenvironment (TPOM) framework [30]. These included technological properties (eg, usability), social dimensions (eg, integration with work practices), organizational dimensions (eg, governance), and system-level influences (eg, policy and markets). Initial versions of the guides were used in early interviews and iteratively refined as data collection progressed to incorporate emerging issues and improve clarity. The guides were also reviewed and discussed within the research team throughout the study to ensure conceptual relevance, consistency across settings, and alignment with the study aims. Participants in the implementation group were interviewed by either JH or CR. In some cases, interviews were conducted with multiple participants together, as requested by those taking part.

**Table 3.** Topic guides interviews.

Theme	Scoping interviews	Baseline pilot site interviews	Follow-up pilot site interviews
Current environment	Current use of ambient AI^a^ tools; user groups; perceived benefits; settings where tools may be useful; potential resistance and reasons; expected outcomes and how to measure them	Current note-taking processes without AI; awareness of documentation practices	—^b^
Implementation	Perceived challenges to adoption; setting-specific issues; mitigation strategies; preparatory work; identification of champions; potential unintended consequences; patient perspectives	Experiences of ambient AI use; impact on care, workflows, and systems; integration with existing systems; unintended impacts	General experience of using [SYSTEM]
Expectations	Expected use of ambient AI in the organization; potential benefits; anticipated challenges; mitigation strategies	Expectations of [SYSTEM] in the pilot site; perceived usefulness; concerns	Comparisons between expectations and actual experience
Documentation time	—	—	Time spent documenting before and after using [SYSTEM]; reasons for changes; impact on referral processes; use of time saved
Interactions	—	—	Changes in interactions with service users and staff
Workplace impact	—	Experiences of system integration; impact on care provision and workflows; unintended impacts	Impact on care provision, workflows, and system integration
Challenges	Perceived risks and concerns about adoption	Concerns arising during early use	Ongoing concerns following implementation
Uptake and attitudes	—	—	Views on wider rollout; changes in perceptions following pilot
Recommendations	—	—	Recommendations for implementation in other settings

a AI: artificial intelligence.

b Not available.

All interviews were audio-recorded and transcribed using AI software (Teams or Word Dictate), with transcripts subsequently checked and corrected for accuracy by the research team (JH and CR) against the original audio recordings. Two clinicians who were unavailable for a follow-up interview provided their responses in written form, submitted to the evaluation team either by email or via a colleague during their own interview. We collected data until no significantly new themes were emerging in the concurrent data analysis (the point at which we achieved thematic saturation). Regular team discussions helped to facilitate this.

### Data Analysis

Transcripts and observation notes were anonymized and coded by the research team (KC, RW, JH, and CR) using NVivo (Lumivero; KC and RW) and Microsoft Excel codebooks (JH and CR). The datasets were analyzed using a mixture of inductive and deductive approaches using thematic analysis to identify recurring patterns and themes across sites and participants. We used the TPOM framework to code the transcripts and to identify to what extent the results aligned with the existing literature on complex digitalization initiatives in health service settings [30].

Emerging findings were discussed among the full research team during regular project meetings, and an analysis workshop involving KC, LMS, RW, JH, and CR was held at the end of the study to synthesize insights from all datasets and develop an overarching thematic narrative. This workshop focused on exploring novel themes that did not fit in with the framework and tensions across TPOM categories. It provided an opportunity to review findings collaboratively and reflect on the implementation journey and lessons learned.

## Results

### Overview

We collected interview data from 45 individuals and 16 hours of observations across 4 locations, including 2 primary care and 2 hospital settings (Tables4  5). In total, 3 of the observations had both baseline and follow-up data, while 1 did not have a follow-up because the system was not regularly used in that setting at the time.

**Table 4.** Overview of interviews.

Interview number	Role	Organization	Data collected
Phase 1: scoping interviews			
1	Vendor	Ambient scribe development or sales	Interviewed twice
2	Manager	NHS^a^ provider organization (region 1)	Interviewed twice
3	Manager	NHS (regions 1 and 2)	Interviewed twice
4	Manager	NHS provider organization (region 1)	Interview
5	Consultant	NHS provider organization (region 2)	Interview
6	Consultant	NHS provider organization (region 1)	Interview
7	Consultant	NHS provider organization (region 2)	Interview
8	Manager	NHS provider organization (regions 1 and 2)	Interview
9	General practitioner	Region anonymized	Interview
10	General practitioner	Region anonymized	Interview
11	Manager	NHS provider organization (region 1)	Interview
12	Manager	NHS provider organization (region 1)	Interview
13	Manager	NHS provider organization (region 1)	Interview
14	General practitioner	Region anonymized	Interview
15	Manager	NHS provider organization (region 1)	Interview
16	Consultant	NHS provider organization (region 1)	Interview
17	Manager	NHS provider organization (region 1)	Interview
18	Vendor	Ambient scribe development or sales	Interview
19	Consultant	NHS provider organization (region 1)	Interview
Phase 2: implementation study interviews and observations			
20	Consultant	NHS provider organization (region 1)	Interview and observation^b^
21	Consultant	NHS provider organization (region 1)	Interview^b^
22	Secretarial staff	NHS provider organization (region 1)	Interview^b^
23	Clinician	NHS provider organization (region 1)	Interview and observation^b^
24	Secretarial staff	NHS provider organization (region 1)	Interview^b^
25	Clinician	NHS provider organization (region 1)	Interview^b^
26	Clinician	NHS provider organization (region 1)	Interview^b^
27	Doctor	NHS provider organization (region 1)	Interview^b^
28	Secretarial staff	NHS provider organization (region 1)	Interview^b^
29	Practice manager	General practice (region 2)	Interview
30	Secretarial staff	General practice (region 1)	Interview
31	General practitioner	General practice (region 1)	Interview^b^
32	General practitioner	General practice (Region 1)	Interview^b^
33	General practitioner	General practice (region 2)	Interview^b^
34	Secretarial staff	General practice (region 2)	Interview^b^
35	Secretarial staff	General practice (region 2)	Interview^b^
36	Practice manager	General practice (region 1)	Interview and observation
37	General practitioner	General practice (region 1)	Interview^b^
38	Secretarial staff	General practice (region 1)	Interview
39	General practitioner	General practice (region 2)	Interview and observation^b^
40	General practitioner	General practice (region 2)	Interview and observation^b^
41	Secretarial staff	General practice (region 2)	Interview and observation
42	Secretarial staff	General practice (region 2)	Interview
43	General practitioner	General practice (region 2)	Interview and observation^b^
44	Secretarial staff	General practice (region 2)	Interview
45	Secretarial staff	General practice (region 2)	Interview and observation

a NHS: National Health Service.

b Participants involved in pilot implementation sites.

**Table 5.** Overview of observations.

Location	Setting	Context	Baseline data collection	Follow-up data collection
Location 1	General practice (large)	Nonmetropolitan English district (population ~57,400)	2 clinics (2 hours; 5 consultations)	Not assessed
Location 2	Adult diabetes clinic	Rural English county (population ~60,475)	1 clinic (2 hours; 4 consultations)	1 clinic (2 hours; 4 consultations)
Location 3	Pediatric clinic	Rural English county (population ~60,475)	1 clinic (2 hours; 3 consultations)	1 clinic (2 hours; 5 consultations)
Location 4	General practice (small)	Rural English town (population ~4700)	1 clinic (3 hours; 3 consultations)	1 clinic (3 hours; 5 consultations)

The study involved several different user groups, including individuals who worked only with the stand-alone system, those who used only the integrated system, and those who had access to both. Participants demonstrated flexibility and enthusiasm toward piloting new ambient scribes throughout the evaluation, despite some challenges encountered. Many described their acceptance that this technology remains in its infancy in terms of clinical utility but acknowledged that there is significant potential for the future.

*I’m sure there will come a time when AI, because the whole job of AI is to make the life of humans easier, not harder. And on this occasion this software made my life harder rather than easier, but I’m sure there will come a time.*

[GP]

At baseline, clinicians also expressed confidence in their general ability to work with new IT systems. However, during the implementation study, some clinicians experienced difficulties using specific functions within the ambient scribe, such as letter generation, and a few discontinued using the system after encountering technical issues.

### Variations Across User Profiles and Contexts

Clear differences emerged between user profiles. Stakeholders’ attitudes toward and experiences with ambient scribe technology varied considerably according to a range of contextual and individual factors. These included clinical setting, specialty, and professional role, as well as the nature and complexity of consultations. Differences in software maturity, customization options, experience and familiarity with particular systems, cognitive processing styles, duration of use, and overall technological confidence also influenced how stakeholders perceived and engaged with ambient scribe solutions.

Clinicians and medical secretarial users who had prior experience with the stand-alone ambient scribe generally preferred it to the integrated solution when comparing the 2. In contrast, users who adopted the integrated system during the pilot tended to be more optimistic about its potential, although some primary care staff discontinued use before the end of the study. This discontinuation was largely attributed to the system’s limited functionality, textual inaccuracies, and difficulties modifying prompts and templates. Such changes on the integrated system required supplier validation and testing, leading to delays and additional demands on users’ already limited time. Many clinicians reported spending substantial time between consultations, filtering out extraneous detail from the AI-generated consultation summaries, restructuring summaries, and adding in additional information that the AI-generated summary had missed. This included medication dosages or pertinent aspects of patient history (medical or nonmedical) or information that was not verbalized but required for notes (eg, prescription numbers and reimbursement codes). Lack of information in the system increased the burden of configuring notes, as relevant information had to be cut and pasted into the EHR, and was difficult to substantially reconfigure within the ambient scribe itself. Configuration was undertaken both by clinicians after consultation and secretarial staff, with time spent on these configuration activities found to offset the productivity gains the system was intended to deliver.

*It takes you 5 minutes to read through what happened in the last consultation, when it would normally take me in 20 seconds because I would just have looked at my brief notes.*

[GP]

These initial setup costs could potentially be outweighed by longer-term benefits; however, the short duration of the pilot and small number of participants prevented assessment of such effects. Respondents reported only modest efficiency improvements from using the integrated system and found it difficult to quantify time or effort saved due to other confounding factors. While none of the participants supported a full roll-out of the integrated ambient scribe system at this stage, most maintained a cautiously optimistic outlook about its future potential.

*Yeah, it did save a bit of time, yeah. But not lots. That was frustrating because I could see that it could save time, but yeah, we just haven’t got to that point where it was good enough.*

[Secondary care clinician]

### Key Themes Emerging Across Stakeholders and Settings

#### Overview

**Figure 1.**
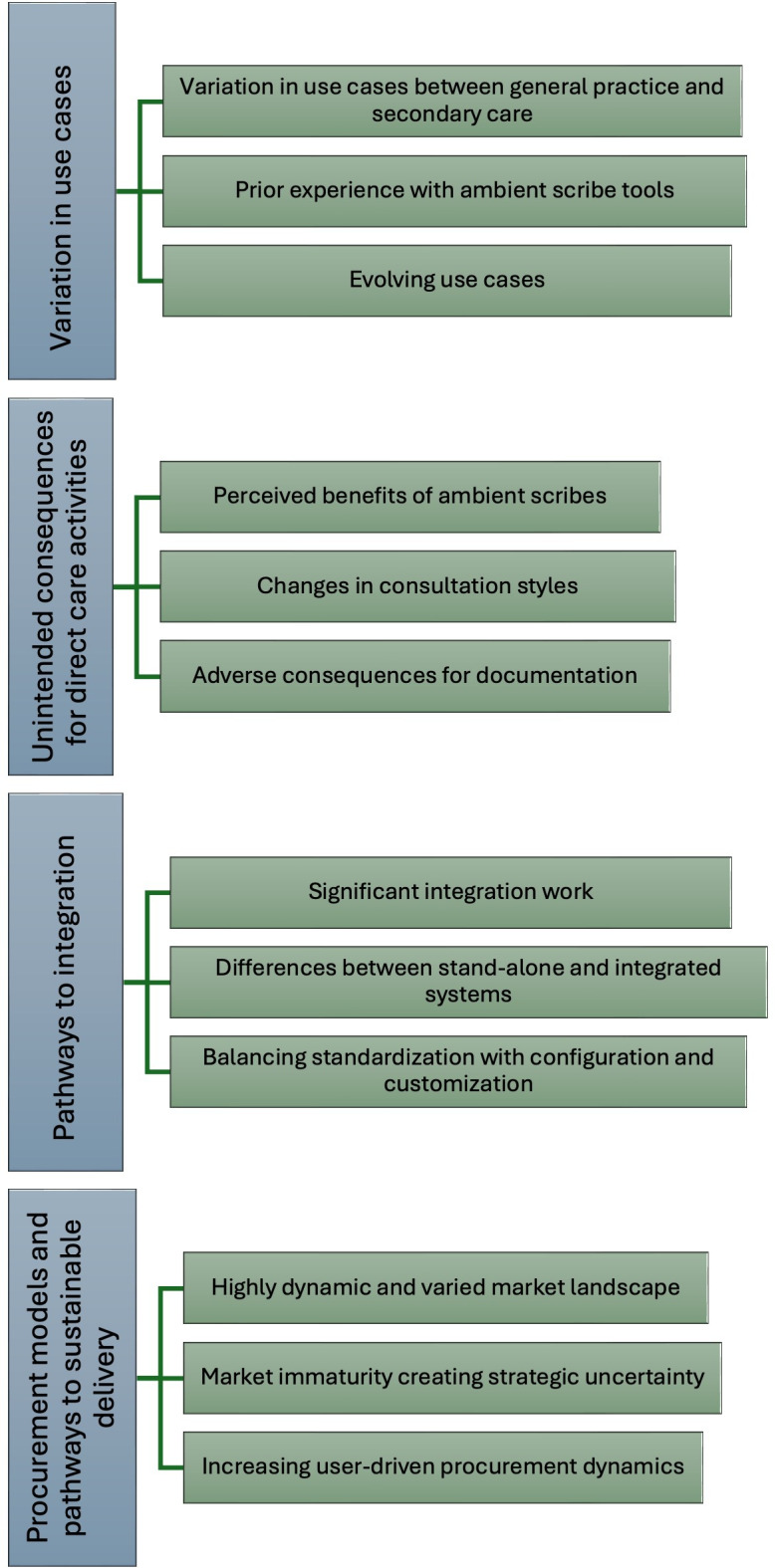
Thematic map of emerging themes and subthemes.

Several cross-cutting themes emerged across the respondent group (Textbox 2, Figure 1, and Multimedia Appendix 1). These are discussed below in detail in turn.

Textbox 2. **Themes emerging from the analysis.**

**Variation in use cases strongly influenced how the systems were deployed in practice**

- Use cases varied substantially between general practice and secondary care
- The purpose of the generated letters and reports differed by setting and care pathway
- The wide range of clinical needs and pathways presented a challenge for developing and validating variants (templates and prompts) for specific users or uses
- Summary and documentation practices differed across settings and between individual practitioners
- Ambient scribe technology is flexible, allowing use cases to be extended

**Unintended consequences emerging within direct care activities**

- Enhanced patient interaction from automating note-taking
- To get the ambient scribe to capture their thinking, clinicians learned to adopt different ways of speaking during the consultation
- Effects on reporting—generic style with loss of personal tone and individual clinical report preferences
- Supported, and in some cases increased, tendencies toward defensive documentation
- Risk of more mechanistic communication
- Reduced immersion when reviewing summaries (ie, summaries not taking clinicians back to the context they were created in)

**Pathways to integration and their associated risk-benefit profiles**

- The stand-alone system had lower adoption and learning costs than the integrated system
- Integration required significant development effort but held out greater promise for improved long-term efficiency and safety
- Integration required standardization and stabilization that was in tension with the diversity of use cases and pathways, and required strategies for addressing diverse contexts including more mature products that could cater for managed diversity through configuration rather than customization
- Tension between standardization, configuration, and customization
- Vendors require more sophisticated strategies to address diverse contexts

**Procurement models and pathways to sustainable delivery within a volatile market environment**

- Highly dynamic and varied market landscape
- Market immaturity creating strategic uncertainty
- Increasing user-driven procurement dynamics
- Heterogeneous implementation of diverse tools likely
- Risks associated with aggressive sales tactics
- Strategic choices for health systems

#### Variation in Use Cases Strongly Influenced How the Systems Were Deployed in Practice

##### Variation in Use Cases Between General Practice and Secondary Care

Use cases for ambient scribe technology differed markedly across care environments. General practice and secondary care exhibited distinct workflows, and significant variation was also evident between secondary care specialties such as pediatrics and diabetes. The purpose and structure of letters and reports generated through the system varied by care pathway and clinical setting, which further shaped how the technology was applied. For instance, general practitioners (GPs) needed to tackle a large range of requirements for referrals to different services, while secondary care consultations were more focused.

These diverse requirements created challenges for standardized system functionality when integrating ambient technology outputs with standardized systems like EHRs. Adapting a single solution to reliably support multiple, highly variable pathways introduced substantial cost and complexity. Medical secretaries continued to play a critical role in managing correspondence and ensuring quality. Documentation practices also differed. GPs were typically accustomed to taking real-time notes directly into their electronic patient record systems during their typically brief patient consultations, and most had developed strong manual note-taking skills, with a focus on accuracy and brevity.

*Time taken to make notes and dictate letters minimal and smooth process*.

[Researcher notes, primary care, baseline observation, location 1]

##### Prior Experience With Ambient Scribe Tools

Though very confident with technology and learning new systems, most participants lacked prior experience with ambient scribe tools. As a result of high adoption costs and marginal benefit over existing practice, they experienced little incentive to transfer. More secondary care clinicians had prior experience with ambient scribes, though not all did, and hospital clinicians generally sought to capture greater detail when documenting consultations than in routine primary care.

*The process of dictating a letter is the process of thinking about the facts, reading the facts, remembering the case, and then distilling that into a narrative [...] there is a process of filtering it all through your brain and then hitting the paper ...*

[Secondary care clinician]

*Less experienced colleagues will just write loads, and they’ll wind up with paragraphs of information. And then people who are a bit longer in the tooth will say, you know, “saw patient, patient is fine,” just a very little, just a couple of lines.*

[GP]

##### Evolving Use Cases

There was also a recognized risk that use cases would evolve over time, with users and organizations finding new ways to use ambient scribe functionality as well as with improving the performance of increasingly mature solutions. This dynamism added another layer of uncertainty for procurement, coordinated roll-out and optimization, and long-term sustainability of system design.

### Unintended Consequences Emerging Within Direct Care Activities

#### Perceived Benefits of Ambient Scribes

Respondents identified several themes related to the impact of ambient scribe technology on the consultation encounter, including the cognitive and interpersonal aspects. Many noted positive effects, including enhanced patient engagement, improved eye contact, reduced mental and physical distraction, and increased transparency during clinical interactions.

#### Changes in Consultation Styles

A range of subtle changes in consultation style and reporting practices were also observed. We briefly summarize these changes here, but addressing these systematically would require a different kind of (larger scale, longer term) study. At baseline, several GPs and secondary care consultants referenced their need to document consultations with accuracy and sufficient detail to provide medico-legal protection in the event of a case being raised. At follow-up, several of these practitioners reported that they felt a benefit of the ambient scribe was its capacity to capture detail that supported this defensive note-taking. Some clinicians reported that ambient scribe–generated notes frequently contained inaccuracies. To improve the accuracy of documentation, they described modifying their communication style to be more structured and explicit during consultations. Some expressed concerns that they might be talking in a somewhat exaggerated or artificial way (“like a parrot”) to ensure that the ambient scribe accurately picked up their words. Similarly, concerns were raised about how well the clinician’s “authentic voice” was sustained within written records:

*I tried to make it very clear, as if there was a ghost in the room, that this is what I’m doing now [...] Yeah, it made me become a bit more robotic ...*

[GP]

*Clinician is speaking to both ambient scribe and patient somewhat mechanically at times*.

[Researcher notes, hospital outpatients, location 2]

#### Adverse Consequences for Documentation

Some participants also noted that the system struggled to capture the clinical reasoning behind their questions. Several clinicians reported adapting their communication style during consultations to make their thought processes clearer—both for the scribe and for the patient. Respondents noted that using the ambient scribe required them to verbalize their observations more explicitly during the consultation to ensure that emerging clinical assessments were captured accurately by the system. Views on this requirement varied. Some clinicians felt that articulating their reasoning aloud reinforced good consultation practice and clinical clarity, while others experienced it as disruptive, altering how they naturally communicated with patients. We also observed variation in clinicians’ acceptance of the generated consultation summaries. Some were satisfied with the writing style, particularly valuing the system’s ability to produce summaries tailored to different levels of medical literacy. Others, however, felt that the output did not align with their preferred documentation style and required additional editing.

When reviewing generated summaries, a few users found that subsequent reading of the automated summaries—unlike their own notes and summaries—did not prompt recall of the earlier patient consultation and diagnosis and so would likely be less facilitative for later consultations.

*So, you know, I have a narrative to share and that narrative I won’t physically remember. I need to be triggered by the letter so I don’t know if the AI generated letter will do that.*

[Secondary care clinician]

### Pathways to Integration and Their Associated Risk-Benefit Profiles

#### Significant Integration Work

Integration of the ambient scribe technology required substantial effort by the vendor and health professionals. Significant preparatory work was necessary to develop and validate templates and prompts, followed by continuous refinement through user feedback. Aligning the solution with existing organizational information infrastructures introduced unanticipated challenges. Integration work encompassed not only the electronic patient record but also documented management middleware, patient communication tools, electronic notes, and discharge summary systems. The lack of standardization across hospital service order processes and referral systems (varying between specialisms and clinics) amplified the complexity of achieving reliable integration. This was a particular challenge within primary care, where secretarial staff navigate referrals to many different external services with specific templates and documentation requirements. Primary care staff were also concerned that medical documentation included the correct terms and details required for quality and outcomes framework (a system used in UK general practice to reward GP surgeries for delivering high-quality care) coding, a feature likely to be key for wider adoption of such systems into primary care.

*There could be [50 types of forms] if we were to count every single one, for the different conditions, types of referrals, everything. Every different part of the body, for cancer, it’s got a different form [...] It would really help in that sense if it could summarise the notes and then fill in the form.*

[Medical secretary, primary care]

#### Variations in Distribution of Set-Up Effort, Adoption Costs, and Benefits of Use

The distribution of set-up effort, adoption costs, and use benefits also differed sharply between integrated and stand-alone systems. Stand-alone solutions delivered immediate, user-level gains by automating discrete tasks, although this placed responsibility for workflow alignment on the individual to check and paste summaries into reports and letters.

*Clinician manually copies and pastes medical history, current medications and test results from [electronic health record system] into the ambient scribe record. This is due to non-integration of the two systems*.

[Researcher notes, hospital outpatients, baseline observation, location 2]

In contrast, integrated systems offered the prospect of longer-term organizational efficiencies but required substantial upfront investment and broader transformation. The short duration of the pilot meant that these longer-term benefits could not yet be observed, and respondents overall reported no material time savings during the study period.

*It’s [integrated system] going to take ... at least three months to set up, test, embed ... whereas if it’s not integrated, you could set that up quite quickly. Obviously in the long term ... it is much more beneficial to have an integrated AI ... integration is more complicated, and it takes longer ... So if you want a quick win, you’re not going to integrate, but your long-term strategy would be that you would integrate your AI, but it takes longer.*

[Manager]

#### Balancing Standardization With Configuration and Customization

A recurring issue involved balancing standardization with configuration and customization. Users appreciated the flexibility to tailor templates in the stand-alone system, whereas the standardized templates required for integration were perceived as restrictive. Vendor validation and approval processes for modifications in the integrated system added further delays because templates had to function consistently across multiple settings. Provider organizations therefore needed to strike a careful compromise that protected interoperability without undermining user engagement and adoption.

*... where there was a bit of a bottleneck was the prompt ... It’s the prompt that tells you that tells the machine what to put on the page and the clinicians kept wanting to tweak their own prompts, while the [provider organisation] said ‘no, you’re not allowed to do that because we haven’t signed off on what you’re putting in your letters from this AI tool’. So, I think the clinicians were getting frustrated quite a bit. They have to like go through us to make those little changes, so we can reuse some prompts, but generally each specialty is gonna require different information in a letter.*

[Supplier]

Vendors may need to develop more advanced strategies to respond effectively and efficiently to diverse clinical contexts and requirements. A model of channeled customization was viewed as a promising approach, involving a curated library of validated templates with controlled scope for local adaptation through configuration (change within predetermined parameters) rather than customization. This approach would help cater for user or use diversity in a managed way while avoiding the extremes of overstandardization or of complete variability, offering users a structured but flexible set of options to support documentation practices.

*What ideally you would probably want is to have some agreed templates for specialties or disciplines with a level of local editability that would appease clinician requirements. So, you kind of want it on two levels. You want to have ... the [provider organisation] going ... we’re going to have two templates for each specialty, for example, or three templates for each specialty. And within that we’ll give the clinicians this much editability to hone those templates for the requirements of specialist clinics.*

[Manager]

### Procurement Models and Pathways to Sustainable Delivery Within a Volatile Market Environment

#### Highly Dynamic and Varied Market Landscape

The market for ambient scribe technologies is highly dynamic and diverse. It currently comprises a mix of numerous recent start-ups and more established systems that have benefited from longer periods of optimization. Solutions are evolving rapidly, with continuous improvements in performance and integration capabilities.

*... that’s like, Whack-A-Mole to me, that we’ll just spend our entire lives just trying to work out who’s coming to market. And they’re literally coming up out of the woodwork, like, Whack-A-Mole. You know, every day I hear of two or three extra.*

[Manager]

#### Market Immaturity Creating Strategic Uncertainty

This immaturity in the market contributes to strategic uncertainty for adopting organizations. Many are hesitant to lock themselves into a single solution because technology continues to change at a pace. Integrated products are especially affected, since they entail more complex implementation and higher switching costs. As a result, procurement processes must become more agile, and deployment strategies need to support coordinated upgrades and structured sharing of local learning across sites.

#### Increasing User-Driven Procurement Dynamics

Procurement is also increasingly being driven by users rather than by organizations. Vendors frequently target individual clinicians or clinical teams directly, sometimes offering free access to encourage familiarity, dependency, and local adoption. This creates challenges related to oversight, information governance, and regulatory compliance, particularly when solutions are introduced outside formal organizational pathways. In April 2025, during the study period, NHS England issued a mandate that any ambient scribe performing summarization in clinical practice should be classified as a Medicines and Healthcare products Regulatory Agency Class 1 Medical Device, reflecting growing concerns about governance and patient safety. Several clinicians reported using tools acquired for their private practice.

*... it feels a bit on this one like national playing catch up, whereas in other scenarios historically it would be a ... plan, a policy, it would be issued out and you’d be told, you know, you’ve got to be doing it by next date and you might not have thought about it yourself yet. So, this definitely feels the inverse of what we would normally see.*

[Manager]

Given these dynamics, a patchwork of heterogeneous implementations across services, specialties, and regions appears likely. Aggressive sales approaches that bypass organizational governance risk further fragmenting decision-making and complicating future rationalization efforts. Health systems will therefore need to consider whether to permit a hybrid environment that accommodates multiple solutions or pursue more standardized adoption of a single product. Each of these strategic choices presents distinct implications for procurement policy, long-term interoperability, and regulatory management.

*... there is a load of challenges around regional procurement because actually they’ve got so many organisations in place. It’s how do you satisfy everybody’s needs and in theory the needs should be the same. But actually, I think they’re subtly different, particularly when it comes to if you want it to be integrated with your with your EPR [Electronic Patient Record]. I think the suppliers will say they can do it and then the reality will be that they can’t. So yeah, or they’ll be able to do it for some and not others. So, I do think there’s going to be some challenges within that ...*

[Manager]

## Discussion

### Summary of Main Findings

We found overall enthusiasm for ambient scribes, but they were used very differently across health care settings, reflecting diverse clinical workflows and documentation practices. While the technology showed flexibility and promise, it also introduced unintended consequences, from changes in clinician communication styles to more generic reporting and defensive documentation. Integrated systems offered greater long-term benefits compared to stand-alone solutions but required significant effort and careful balancing between standardization and local adaptation.

Overall, this work shows that despite strong enthusiasm and rapid uptake, the deployment of ambient scribes is still at an early stage, and the technology has not reached maturity. Risks and benefits remain uncertain, and effective implementation will require careful planning, iterative learning, and attention to evolving market and system conditions.

### Strengths and Limitations

It is still very early in the evaluation of ambient scribe technologies, but this work contributes to the emerging evidence base. Our study examined real-world adoption processes across different systems and service settings, providing high ecological validity. However, we focused primarily on a single integrated system, while many users had also experience of using a second stand-alone ambient scribe. This may have led to carryover effects from prior learning and direct comparisons with the alternative solution. It may also reflect the features of the system in question and limit generalizability to other integrated systems. Future studies should therefore aim at more direct comparisons between integrated and stand-alone systems, ideally across a range of institutional contexts, as our findings may lack generalizability.

Clinicians in this study were generally confident users of new technology, which supported strong engagement in data collection activities and excellent follow-up response rates. Future evaluations would benefit from involving staff and organizations who are not natural early adopters or who hold significant reservations about ambient scribes. Understanding the needs and behaviors of different user and organizational profiles will be essential for preparing the wider workforce and for designing effective protocols and training packages that accommodate a diverse range of staff. Mitigating against this early adopter bias will also be crucial in identifying adoption challenges and unintended consequences.

The short time frame of this evaluation and the implementation were clear limitations, but it reflects the rapid, real-world deployment of ambient scribe solutions across services. Further research is needed to understand long-term integration with different EHRs. Longitudinal studies are particularly important, as many unintended consequences, workflow adaptations, and adoption patterns may only become visible over the medium to long term, including impacts on clinical follow-up, decision-making, burnout, workload, and patient satisfaction.

We initially aimed to estimate time spent on key activities before and after scribe deployment to understand efficiency impacts. However, participants found it difficult to provide reliable time estimates, and these were self-reported and subjective. While our work provides valuable insights into how staff felt about their experience with ambient scribes, future work must strike a balance between the cost and complexity of rigorous time-and-motion studies (which will inevitably vary across contexts) and the use of simpler, less robust self-reported indicators that are easier to collect and still valuable for informing business cases in rapidly evolving environments.

### Integration of Findings With the Existing Literature

Research on ambient scribes has so far focused predominantly on their impact on clinician efficiency and burnout related to administrative workload [1-4,31]. Our study contributes to this growing evidence base in several important ways.

First, we found that early-stage adoption appears to yield at best moderate improvements in efficiency. These benefits vary substantially across settings, systems, and individual users. While many existing studies report considerable gains in efficiency, workload reduction, and burnout mitigation [9-14,32-35], our findings highlight the need for a more nuanced understanding. Efficiency benefits cannot be generalized without examining contextual factors such as organizational workflows, user characteristics, and how these evolve over time.

Second, our study draws attention to emerging unintended consequences—an area that has received considerably less scrutiny than exploration of impacts [14]. Consistent with previous work [11,13], clinicians described several positive consultation effects, including improved eye contact and reduced cognitive load during consultations. However, we also observed notable shifts in consultation dynamics. Clinicians reported modifying their communication style to “speak to the scribe,” verbalizing thoughts they would not normally articulate during a natural patient interaction. In addition, ambient scribes were found to affect cognitive processes related to note-taking, potentially hindering information retrieval. These issues warrant a more detailed, longitudinal investigation to understand how ambient scribes may reshape clinical work, patient relationships, administrative routines, and documentation practices over time [36,37]. This will also require attention to how the patient voice and even the patient record may be reshaped through algorithmic summarization and template alignment. AI-driven structuring may privilege biomedical categories over experiential accounts, risking narrative elements (which are often crucial in relation to patient experience) to be neglected in structured outputs [38]. In doing so, ambient scribes may subtly reconfigure what counts as clinically relevant, although this may not be in the best interest of the patient and their care.

Finally, our study provides insight into the differing implementation and adoption pathways associated with stand-alone versus integrated ambient scribe systems, including the benefits, trade-offs, and affordances of each. Previous research has not explicitly examined these distinctions; yet, they are likely to become increasingly significant. In the long term, benefit profiles will depend heavily on the quality of integration between ambient scribes and EHRs; in the short term, strong bottom-up user demand is likely to drive adoption patterns [12,13,32,34,39]. Organizations, particularly those without active engagement from their primary EHR provider, may face difficult decisions around standardization, governance, and procurement. These tensions reflect broader debates within medical informatics regarding standardization versus local customization [40]. What is distinctive in the case of ambient scribes is the strong user pull driving ground-level uptake, which is challenging organizational structures to keep pace with oversight and regulation.

### Recommendations for Policy and Practice

Different clinical pathways operate on varying timescales, so strategies must balance short-term usability with long-term integration goals. Stand-alone systems can deliver quick wins for clinicians, while integration remains essential for future interoperability and organizational efficiency. Organizations should avoid entering overly long contracts or buying licenses for all users upfront, instead adopting a measured, flexible approach that allows learning and adaptation as the market evolves.

Early procurement should focus on short-term, limited licenses with a structured rollout, embedding process improvements and evaluating outcomes before committing to wider deployment. This will help to ensure resources are targeted where benefits are likely and support evidence-based decision-making. Evaluation findings should inform procurement, though this remains challenging in a context of rapid, bottom-up adoption and changing vendor landscapes.

Initial deployments should target clearly defined use cases, recognizing that the same technology may be applied differently across clinical settings. Shared learning between sites and vendors will help develop common standards and governance processes, particularly for managing changes to prompts or templates. Maintaining a human-in-the-loop approach is essential to ensure safety and clinical accountability, as there are still many risks associated with unintended consequences of systems (eg, impacts on interactions, hallucinations, and cognitive processing) and significant variations across contexts of use. These often only become apparent once systems are deployed in real-world contexts of use, highlighting the importance of concurrent monitoring.

As adoption progresses, incremental, opt-in deployment is preferable to large, mandated rollouts, allowing systems to mature while minimizing risk. Ongoing workforce education and engagement, delivered by local champions within clinical environments, will be key to building confidence, addressing concerns, and ensuring responsible use of ambient scribe technologies.

We summarize wider policy implications emerging from these dynamics in Textbox 3.

Textbox 3. **Summary of policy implications emerging from this work.**

Strategies must balance short-term local usability and benefits with wider long-term integration and interoperability goals within and across organizations.

There is a need to develop novel procurement strategies that avoid vendor lock-in and allow local experimentation and flexibility in line with evolving markets. These should consider different pathways including stand-alone and integrated systems.

Clearly defined use cases of procurement and roll-out can serve as exemplars or blueprints for wider learning across organizational settings. Identifying lessons learned will be particularly important. These need to be used to incrementally build on local lessons before wider roll-out.

Clarifying governance and accountability implications as well as workforce education will be crucial for the safe implementation and adoption of ambient scribes.

Future research must play a key role in informing these efforts. For example, longitudinal effects of ambient scribes on clinical workflows and documentation practices are currently not known and need to be explored over time, identifying and mitigating emerging risks for patient safety before adverse effects occur. There is further a need to conduct comparative studies between integrated EHR-embedded systems and stand-alone tools to better understand the different pathways and risk-benefit profiles over time. Other areas to explore include impacts on clinician burnout, cognitive demands, documentation practices, and changes in patient-provider interactions and relationships.

### Conclusions

The deployment of ambient scribe technologies remains at an early stage, characterized by strong enthusiasm and rapid experimentation. However, this enthusiasm must be balanced with careful attention to safety, quality, and potential unintended consequences. These systems are likely to evolve substantially as integration with different EHR platforms progresses, offering the prospect of significant long-term benefits for clinical efficiency and documentation quality.

At the same time, it is important to remain mindful of broader system-level effects. Evaluation should extend beyond measuring improvements in individual productivity to include impacts on teamwork, communication, workplace tasks, and roles and organizational dynamics, ensuring that innovation enhances, not fragments, health care delivery.

## Supplementary material

10.2196/88472 Multimedia Appendix 1 Identified themes, subthemes, and illustrative quotes.

## Abbreviations

AI: artificial intelligence
EHR: electronic health record
GP: general practitioner
NHS: National Health Service
TPOM: technology-people-organization-macroenvironment
