# Supplementary material for: The Regional Implementation of an Electronic Health Record–Integrated Ambient Scribe in Primary and Secondary Care in England: Real-Time Qualitative Evaluation
Source: JMIR Med Inform. 2026 May 29;14:e88472. doi: 10.2196/88472 (PMC13220976; doi:10.2196/88472)
Supplement: Multimedia Appendix 1 [file medinform-v14-e88472-s001.docx]

## Table S1. Identified themes, subthemes, and illustrative quotes.

| **Identified theme** | **Sub-theme** | **Illustrative quotes** |
| --- | --- | --- |
| Variation in use cases deployed in practice | Variation in use cases between general practice and secondary care | *With the system that we've got at the moment, you listen to what's being said, and you type it out. So you've got a point of reference. Whereas with the AI system, you don't know if they are saying what [it records] they're saying. You've got nothing to compare it to…I’ve got nothing to listen to and there’s nothing written, apart from what’s been picked up [by the ambient scribe]. So I’m reliant on a computer system that could potentially be picking up the wrong thing.* Medical Secretary  *No manual note-taking at all was undertaken in this clinic, due to the clinician wearing protective gloves during consultation. Clinician talked into [ambient scribe system] during the consultation throughout (while patient was present).* Observation notes, hospital clinic (examination and treatment sessions)  *We're really keen on doing innovative things, [name’s general practice] we're involved in a lot of clinical research and we do a lot of training with medical students and in [name] medical school.* GP |
|  | Prior experience with ambient scribe tools | *Yeah, I've played around a little bit with [standalone system] a little while ago just to see if that was any good or not…I found that it was helpful and generally speaking did pick up things well. I didn't find too many errors in it. Like all AI, occasionally, it will just hallucinate and just make up something that you haven't said, nobody had said, that wasn't even relevant. But that happened very, very infrequently.* GP  *So when I used [standalone system], I think I used the free version, because one of my friends who's subscribed to the full version, you know, was selling me a lot of nice, fantastic things it could actually do. But I was just limited to just transcribing aspects of it.*  GP  *There's lots of trainees who do use it, and they do it without anyone knowing and without asking people. So that's a risk.* GP  *I don't understand how it how it works at all. […] I've got no experience on it whatsoever.* Medical Secretary |
|  | Evolving use cases | *For an AI generated referral, you want the whole thing to be done. You want that referral letter. So, rather than me dictating referral letter, it just regurgitates the referral letter to check through.* Consultant  *Things like family history. So, if I say ‘No family history of ischemic heart disease’, will it pick that out and code it? […] I'm just saying that that's the sort of thing that would be really helpful.* GP |
| Unintended consequences for direct care activities | Perceived benefits of ambient scribes | *I must admit it did feel kind of magical kind of taking what was being said and then turning it into something which I felt mostly accurately represented.* Consultant  *I think the biggest benefit of this is actually not clinician time. It's actually patient experience…you end up typing with the patient so you can't do anything else, so you have to make little notes, so you type. But the patient thinks you're not listening to them. So, the big advantage of this I can focus on the patient I can maintain eye contact, I can listen to them, and I was listening before, but I was listening and typing so I didn't forget it whereas now I can listen…I think that's with some of the magic in this is.* GP |
|  | Changes in consultation styles | *So, it sounds like you're sort of talking the patient through it. But actually, you're talking to the machine, and I say…your chest sounds really clear… your throat looks clear. So you end up talking through the examination. So, if I'm examining somebody's abdomen, I will say, your tummy feels nice and soft… I think it's actually helpful for the patient that they're hearing your sort of thoughts and they think you're doing it for them, but actually you're doing it for the machine.* GP |
|  | Adverse consequences for documentation | *[system] is unreliable for recording drug names – correcting the notes was adding to much time to my workload…* *It was even confusing drugs with rather different sounding names.* Consultant  *… what if Kilimanjaro, gets misspelled? What if there's the wrong postcode? What if you know, the system goes down? What if a clinician closes the web page and those sorts of things? So, it's all to do with mitigations. So again, it's well, how do you control that and stop that from happening? And generally speaking, was there's kind of three layers to it, you either design the system to bake the risk out or reduce its likelihood and its impact, there's, human control, which is generally your weakest control. And then there's a process control as well. So generally, it's about trying to design the system to stop that sort of thing from happening in the first place…* *So very much it's around your post deployment as well. It's not just a case of we've done X y and Z and particularly around ambient scribes. There may well be, some potential risks and hazards we've not thought of yet. So, those experiences and incidents, they are supposed to be, you know, reflected on assessed and then baked into your hazard work. And again new mitigations. Done. So again, it's around that continual life cycle sort of thing as well.* Vendor |
| Pathways to integration | Significant integration work | *There is still some rearranging to do, and rearranging the text in the [ambient scribe] native software is apparently awful, so you can't make tables and you can't get it to look like the [hospital] templates. So people hate that.* Consultant  *System integration I think is absolutely key. If you want general practice, primary care to kind of buy into the system.* GP  *Interviewees reported that more time needed to be given to preparing and testing out templates, using the system collectively, and getting to grips with it before implementing.* Notes from interview with Practice Manager |
|  | Variations in distribution of set-up effort, adoption costs, and use benefits | *We didn't have meaningful face-to-face discussions with [ambient scribe developers] and whenever we did have discussions, it was always about things like how to use the system. We needed discussions where they try to understand our jobs and try to understand what sort of output that we needed. So how were they ever gonna produce something that was gonna really work for us?* Clinician  *I didn't have that time set aside for product development. […] That's why I wasn't really fully able to engage with this trial because my expectation was that I'm using a product, but then it turns into ‘No, you’re actually helping me to make a product and I'm not being paid by [ambient scribe developer]. I don't have that extra time to help keep, and so my engagement suffered because of that.* Clinician  *The initial attitude was, you know, this is coming out and you've gotta come to this training session. My clinics are booked three months in advance. So, you know, I can't come to a training session when I've got patients sat in the waiting room. And there was an attitude […] that you’ve got to, you need to, you're obliged to. That's all well and good, still, I can't.* Clinician |
|  | Balancing standardisation with configuration and customisation | *I think I know the [standalone system] people because I've been on a few of their talks. They're not really pushing integration too hard. I think they realise if they push hard on integration …then they're not going to get their product out to lots of people.* Consultant  *You have got the templates that they supply. You can [also] customise the templates. You can enter it here, what you want it to do. So I told it, `this is what I want you to make note of, this is the type of consultation’. I specifically said a structured consultation for a mental Health Review appointment, so it knows the context. So when you go into a menu, when you've got a, a consultation regards mental health, you can go to that customised template and you can generate the consultation using that […] that is useful.* GP |
| Procurement models and pathways to sustainable delivery | Highly dynamic and varied market landscape | *…it feels a bit on this one like national playing catch up, whereas in other scenarios historically it would be a .. plan, a policy, it would be issued out and you'd be told, you know, you've got to be doing it by next date and you might not have thought about it yourself yet. So this definitely feels the inverse of what we would normally see from that.* Manager |
|  | Market immaturity creating strategic uncertainty | *I said, well, that's like, Whack-A-Mole to me, that we'll just spend entire lives just trying to work out who's coming you to market. And they're literally coming up out of the woodwork, like, Whack-A-Mole. You know, every day I hear of two or three extra.* Manager |
|  | Increasing user-driven procurement dynamics | *…so we are going to want to procure some tools now for a number of users that actually we might change the number of users we might decide we don't want to use that tool anymore or we might move within 6 to 12 months. So, I think it's going to test some of those contractual arrangements in terms of having a much more agile and flexible approach to compliant contracts for digital.* Interview Manager |
